# Pharmacokinetic analysis of the soluble guanylate cyclase activator cinaciguat (BAY 58-2667) in individuals with renal impairment compared to healthy controls

**DOI:** 10.1186/1471-2210-11-S1-P61

**Published:** 2011-08-01

**Authors:** Christian Scheerans, Martin Blunck, Wolfgang Mueck, Reiner Frey

**Affiliations:** 1 Bayer HealthCare AG, Wuppertal, Germany

## Background

Cinaciquat (CIN) is a guanylate cyclase (sGC) activator that induces cyclic GMP generation and vasodilation preferentially in diseased vessels [1]. CIN has the potential to increase cardiac output in patients with acute decompensated heart failure [2]. CIN is predominantly and rapidly cleared by the liver [3], and thus, it was not expected that the kidney function would have an influence on CIN clearance. Nevertheless, we determined the pharmacokinetics of CIN in individuals with renal impairment and age- and gender-matched healthy volunteers with normal renal function [4].

## Material and methods

In this non-randomized, non-blinded study, individuals were grouped on the basis of their creatinine clearance (CL_cr_) obtained from a 24 hour urine collection:

• Group 1 (healthy control): CL_cr_ > 80 mL/min

• Group 2 (mild renal impairment): CL_cr_ 50 - 80 mL/min

• Group 3 (moderate renal impairment): CL_cr_ 30 < 50 mL/min

• Group 4 (severe renal impairment): CL_cr_ < 30 mL/min (not on dialysis).

34 individuals received a single 4 hour infusion of 400 µg CIN at a constant rate of 100 µg/h. Plasma concentrations of CIN were determined by high performance liquid chromatography with mass spectrometry. Pharmacokinetic parameters were calculated by noncompartmental analysis in WinNonlin Professional (version 4.1.a).

## Results

There was no evidence of increased drug exposure in individuals with renal impairment. The apparent volume of distribution at steady state (V_ss_) was slightly increased in individuals with renal impairment (Table 1). Moreover, the total body clearance (CL) from plasma tended to increase with progression of renal impairment, which can be explained by an increased hematocrit in individuals with renal impairment, considering the fact that CIN does not distribute in blood cells. The resulting terminal half-life (t_1/2_) in individuals with renal impairment was comparable to the value found in healthy controls. The fraction of CIN unbound (f_u_) in plasma was very low (< 1%) in all groups, and tended to increase with progression of renal impairment, which might be caused by a decrease of albumin concentration. The incidence of adverse events (mostly mild) was similar in all groups.

**Table 1:** Pharmacokinetic parameters of CIN in healthy controls and individuals with mild, moderate and severe renal impairment, showing values as geometric mean (coefficient of variation, %) except for fu which is expressed as arithmetic mean (standard deviation).

Renal function group	CL (L/h)	V_ss_ (L)	t_½_ (h)	f_u_ %
Group 1: Healthy control (n=9)	36.12 (12.3)	18.70 (19.2)	1.56 (53.4%)	0.45 (0.12)
Group 2: Mild renal impairment (n=8)	41.43 (15.3)	26.56 (37.6)	1.72 (65.1%)	0.42 (0.07)
Group 3: Moderate renal impairment (n=9)	40.58 (18.9)	24.02 (27.1)	1.58 (49.6%)	0.53 (0.15)
Group 4: Severe renal impairment (n=8)	51.81 (26.2)	23.51 (18.7)	1.08 (64.0%)	0.66 (0.16)

## Conclusion

The pharmacokinetic results are similar to previous results observed in healthy young men [3]. The CL of CIN is dependent on hematocrit, which is reduced in renal impairment. However, as hematocrit changes are limited in magnitude (i.e. 0.3-0.5), the effect is on CL is also clearly limited. Thus, no dose adjustment of CIN is recommended for renal impairment.
